# Clinical efficacy of three types of autogenous bone grafts in treatment of single-segment thoracic tuberculosis: A retrospective cohort study

**DOI:** 10.7150/ijms.47309

**Published:** 2020-10-16

**Authors:** Ke Tang, Jianxiao Li, Tianji Huang, Weiyang Zhong, Xiaoji Luo, Zhengxue Quan

**Affiliations:** Department of Orthopedic surgery, The First Affiliated Hospital of Chongqing Medical University, Chongqing, China.

**Keywords:** bone graft, thoracic spine, spinous process, lamina, transverse process strut, tuberculosis

## Abstract

A retrospective study investigated and compared the results of lamina with spinous process (LSP), transverse process strut (TPS) and iliac graft (IG) as bone graft in thoracic single-segment spinal tuberculosis(TB) with the one-stage posterior approach of debridement, fusion and internal instrumentation. 99 patients treated from January 2012 to December 2015 were reviewed. LSP was performed in 35 patients (group A), TPS was undertaken in 33 patients (group B), and IG was carried out in 31 patients (group C). Surgical time, blood loss, hospitalization time, drainage volume, and follow-up (FU) duration were recorded. The visual analog scale (VAS), Oswestry Disability Index (ODI), erythrocyte sedimentation rate (ESR), C-reactive protein (CRP), American Spinal Injury Association (ASIA) grade, segmental angle, intervertebral height and bone fusion time were compared between preoperative and final FU. All the patients were followed up for a mean 43.90±10.39 months in group A, 45.30±6.20 months in group B, 44.32±7.17 months in group C without difference(*P*>0.05). The mean age was younger, the blood loss was less, the hospitalization time and the surgical time were shorter in group A than those in group B and C (*P*<0.05). The drainage volume was less in group A than that in group B and group C. The CRP, ESR, VAS, and ODI were significantly decreased and there were no significant difference among the groups at the final FU. The neurological function after surgery was improved compared with preoperation among the groups. The bony fusion at a mean time 12.90±3.91 months in group A was longer than that in group B (6.75±1.55 months) and group C (5.52±1.64 months) (*P*<0.05). No significant difference was found at the mean segmental angle, mean intervetebral height of preoperation and final FU among the groups (*P*>0.05). In conclusion, the LSP and TPS as bone graft are reliable, safe, and effective for single-segment stability reconstruction for surgical management of thoracic TB and TPS could be new bone graft methods.

## Introduction

The spine tuberculosis (TB) is high risky, resulting in bone destruction, spinal deformity, and/or even paraplegia which should be paid more attention. Usually, anti-TB drugs, surgery and supportive managements are considered as effective treatment for spinal TB 1,2. When the patients are indicated for surgery, the radical lesion debridement, solid fusion of implants and standard course of anti-TB drugs are considered successful treatment of spine tuberculosis. Among the surgical management, the bone grafts play the key roles in curing spinal TB which could rebuild spinal stability and maintain the alignment 3-5. Currently, the autologous iliac crest, allogeneic bone graft, rib graft, fibula and titanium mesh cages are reported widely, which are associated with longer surgery time, massive surgical blood loss or cages subsidence, while transverse process and lamina with spinous process(LSP) as bone grafts have rarely been reported.

To achieve the sufficient support strength and a satisfactory fusion rate, we reported two new bone grafting methods to minimize donor site complications through a posterior-only approach debridement and internal instrumentation in treatment of thoracic single-segment spinal TB, comparing the clinical efficacy with iliac crest, to let patients recover quickly after operation.

## Materials and Methods

### Patient selection

The Institutional Review Board of our hospital approved this study and conducted in accordance with the Declaration of Helsinki. All participants provided written informed consent before their data were stored in our hospital database and used for study purposes. From January 2012 to December 2015, in the spine unit of the department, a total of 99 patients with thoracic single-segment spinal TB were reviewed retrospectively and weredivided into three groups. When communicating with patients before surgery, the advantages and disadvantages of the three types of bone grafts managements were fully introduced, so the patients could choose the right treatment for themselves. The surgery procedure was performed by the same spine team. The inclusion criteria were as follows: adult thoracic single-segment spinal TB, one-stage posterior approach, internal instrumentation and reconstruction, and the patients were indicated for surgery: increasing kyphosis, neurological deficits, bone destruction affecting the stability. The exclusion criteria were as follows: active pulmonary TB, extrapulmonary TB, trauma, spine tumor, and spinal metastasis.

### Preoperative management

Anti-TB Chemotherapy was administered as soon as the diagnosis was suggested clinically. Anti-TB drugs with the HREZ standard regimen that consists of isoniazid (5-10 mg/kg/day), rifampicin (10 mg/kg/day), ethambutol (15 mg/kg/day), and pyrazinamide (25 mg/kg/day) was administered 3-4 weeks before the surgery 2,10. Surgical treatment was required when the erythrocyte sedimentation rate (ESR), C-reactive protein (CRP), decreased obviously to normal, or near-normal.

### Surgical procedure

After the administration of general anesthesia, the patients were placed in the prone position. The posterior spinal elements including the lamina, facet joints, and transverse processes were exposed through a midline incision. The pedicle screws of four-level or five-level were fixed based on imaging and C-arm X-ray findings, which were used to ensure its accuracy. The entire LSP or TPS was cut off using a sharpened drill or ultrasonic osteotome, and then one of the facet joints was removed for decompression and radical debridement. The LSP or TPS was trimmed by a bone knife, a sharpened drill, or an ultrasonic osteotome to obtain a suitable and three-cortical-sided bone graft. Decompression and radical debridement were performed. The LSP, TPS or IG were cut off and the area was trimmed to a suitable bone graft. According to the space remaining after radical debridement, one was implanted and appropriately forced with instrumentation locking. Streptomycin 1.0 g and isoniazid 0.2 g mixed with gelatin were used locally. Negative pressure drainage was managed postoperatively, and the specimens were sent for bacterial culture and pathology.

### Postoperative care

The patients continued the oral HREZ chemotherapy postoperatively. One year later, the oral pyrazinamide was stopped and the patients continued 6-month regimens of HRE chemotherapy (12HREZ/12-18HRE). Rehabilitation therapist-guided ambulation exercise was started at 1 week after operation. All patients were evaluated clinically and radiologically at 1 week, 3 months, 6 months, and 12 months after the operation and annually thereafter.

### Follow-up index

For all patients, the data were observed perioperatively and during follow-up (FU): (1) the operation time, surgical blood loss, hospitalization time, drainage, FU time and bone fusion time. (2) Segmental angle. According to the Cobb method, the segmental angle was defined as the angle formed between the superior endplate of the upper vertebral body and the inferior endplate of the lower vertebral body. (3) Intervertebral height is defined as the vertical height between the upper and lower vertebral bodies of the fused segment on the lateral X-ray. (4) Neurological function assessed by American Spinal Injury Association (ASIA) grade. (5) VAS and ODI (6) ESR and CRP. The bone fusion was assessed using criteria of Bridwell et al. 6-7 with the X -ray and CT while necessary. All radiographic data and measurements in this study were reviewed by one senior spine surgeon and one senior radiologist.

### Statistical analysis

The statistical analysis was performed using Statistic Analysis System (SAS Institute Inc., Cary, NC, USA). The results are expressed as mean ± SD. Differences with *P* values < 0.05 were considered statistically significant.

## Results

### Clinical assessments

All the patients were followed up for a mean 43.90±10.39 months in group A, 45.30±6.20 months in group B, 44.32±7.17 months in group C without difference (*P*>0.05). The mean age was younger, the blood loss was less, the hospitalization time and the surgical time were shorter in group A than those in group B and C (*P*<0.05). The drainage volume was less in group A than that in group B and group C (**Table 1**). The CRP, ESR, VAS, and ODI were significantly decreased there were no significant difference among the groups at the final FU except the ESR was higher in group A than that in group B and group C (**Table 2**). The neurological function after surgery was improved compared with preoperation. In the group A, 2 patient improved from grade B to grade D, 5 patients from grade C to grade D, 8 patiens from grade D to grade E. In group B, 15 patients improved from grade C to grade D and 5 patients from grade D to grade E. In group C, 10 patients improved from grade C to grade D and 3 patients from grade D to grade E. There was no significant differences in ASIA grades before surgery and at final FU among the three groups (*P*>0.05).

### Radiological assessments

**Figure 2:**
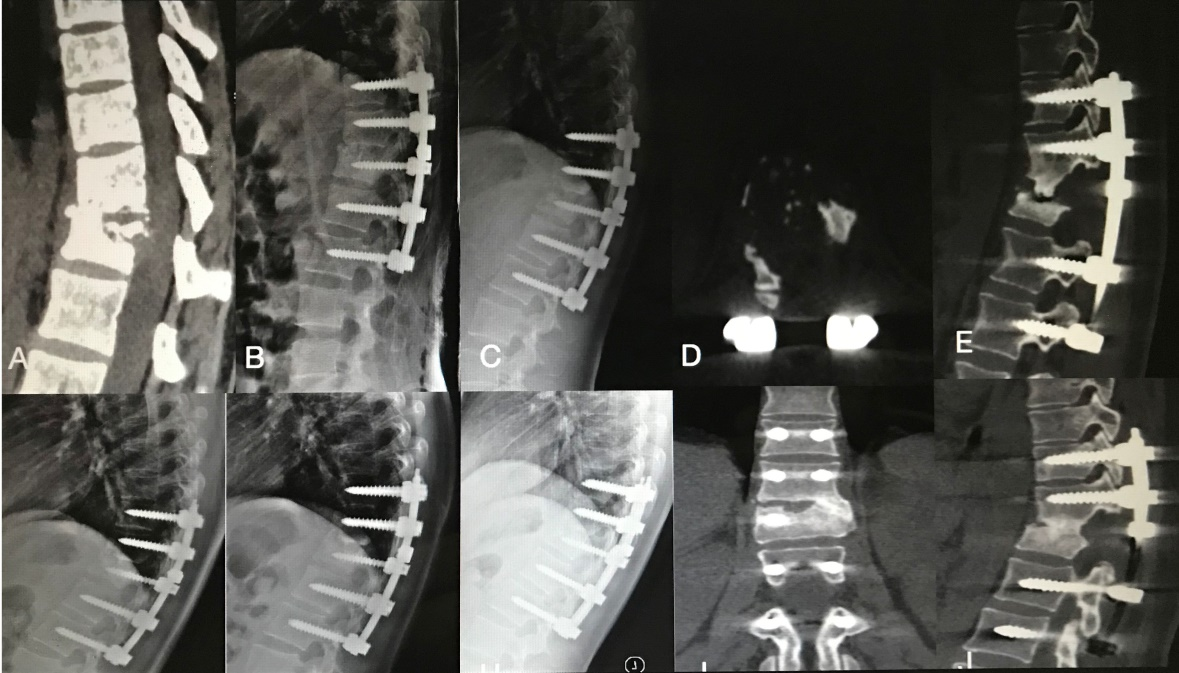
An 22-year-old female patient with thoracic spinal tuberculosis (T11-12) underwent posterior debridement and decompression combined with instrumentation. (A) Preoperative computed tomography (CT) showing bone destruction of the T11-12 vertebrae and compression of the spinal cord. (B,C,D,E) 1-week and 6-month postoperative X-rays and 6-month postoperative CT showing maintained correction and the bone wasn't fully healing. (F,G) 1-year and 2-year postoperative X-rays showed kyphosis correction have loss. (H,I,J)At 4.5-year follow-up, plain X-ray, and CT show solid bone fusion.

**Figure 3:**
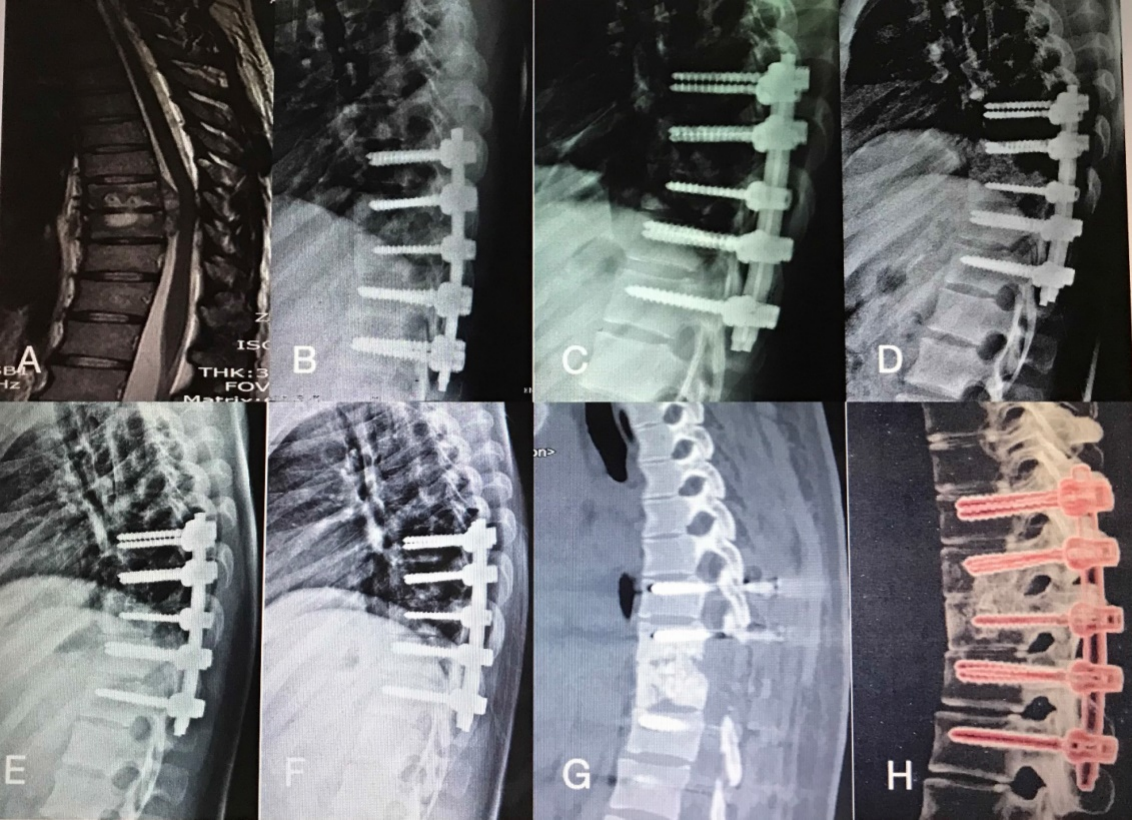
A 45-year-old man with thoracic spinal tuberculosis (T9-10)underwent single-segment posterior debridement and decompression combined with internal fixation. (A)Pretreatment MRI showing the destruction of the T9-10vertebrae and concomitant compression of the spinal cord. (B,C,D,E) Immediate, 3-month, 6-month, 1-year, 2-year postoperative X-ray demonstrated good location of bone graft and internal fixation. (F,G,H) At the 36-month follow-up, plain X-ray and CT showed maintenance of the correction and solid fusion.

The thoracic spinal TB was well cured and the bony fusion at a mean time 12.90±3.91 months in group A was longer than that in group B (6.75±1.55 months) and group C (5.52±1.64 months) (*P*<0.05) (**Figures 2 & 3**). No significant difference was found at the mean segmental angle, mean intervetebral height of preoperation and final FU among the groups (*P*>0.05).

### Complications

Some postoperative complications occurred, such as water electrolyte imbalance (one cases in group A and three case in group B), superficial infection of iliac donor sites (two cases in group C) which were healed by dressing changes and oral antibiotics.

## Discussion

TB is a common extrapulmonary form of an ancient disease and according to the World Health Organization (WHO), TB causes 1.81 million deaths in Asia and China reports 78% new cases annually 1-3. Human immunodeficiency virus (HIV) coinfection, bacterial resistance, childhood tuberculosis and population migration have caused the resurgence of all forms of TB 4-5. Spinal TB is the most common and severe form of osteoarticular TB. Usually, formal adequate and long-term anti-TB drugs, surgery and supportive managements are considered as effective treatment for patients affected by bone destruction, sequestration, a paraspinal or spine canal abscess, or nerve impairment. Surgery via a posterior pedicle screw system with bone grafting and correction of the kyphotic deformity has been widely used for treating thoracic or lumbar spinal TB 8-11.

After complete lesion debridement, many interbody bone grafts are used to restore anterior and middle column stability. Titanium mesh cages filled with autograft have been extensively used and have high bone fusion rates. However, the problems of subsidence, stress shielding, and radio-opacity affect surgical plans 12-13. Thus, the current study aimed to identify new bone grafting method that can provide strut support and bone fusion to decrease the complication rate 14-15.

The use of LSP and TPS as the bone graft has the following advantages. First, compared with the iliac graft, the LSP and TPS could reduce trauma and bleeding; shorten surgical, decrease postoperative drainage volumes, and postoperative complication rates. The VAS and ODI were improved significantly at final FU. Second, in our study, all patients achieved the bony fusion at a mean time 12.90±3.91 months in group A was longer than that in group B(6.75±1.55 months) and group C(5.52±1.64 months). Although the LSP gained longer time of bone healing, no significance was found between the TPS and IG. At the correction of segmental kyphosis, there was no significance among the groups. Hence, the LSP and TPS could provide good support, strength, and fusion properties, and the bony fusion time of TPS was nearly the same with IG. Furthermore, LSP and TPS, as an autogenous bone, have a cortical bone structure supporting bone defect space, and could ensure and maintain the segmental stability and alignment. And comparing with LSP, the TPS could achieve shorter bony fusion time and good clinical results, which could be recommended as bone graft for treatment of single-level thoracic TB 16-17.

However, we declare that the retrospective nature of the small-sample study may be associated with bias. Second, single LSP as bone graft had long bony fusion time and may be risk for delay of the bony fusion. Third, the study didn't consider the intra- and inter-observer differences and biases. Fourth, the biomechanical testing of human cadaver spines will be verified to ensure the strong support. In the future, the prospective, randomized studies with long-term follow-up periods are needed.

## Conclusion

Our study results showed that use of the LSP or TPS could be one choice in the surgical management of single-segment thoracic spinal TB, resulting in good bone fusion and spinal stability restoration, is a reliable, safe, and effective bone grafting method. Furthermore, the TPS could be a new bone graft method.

## Figures and Tables

**Figure 1:**
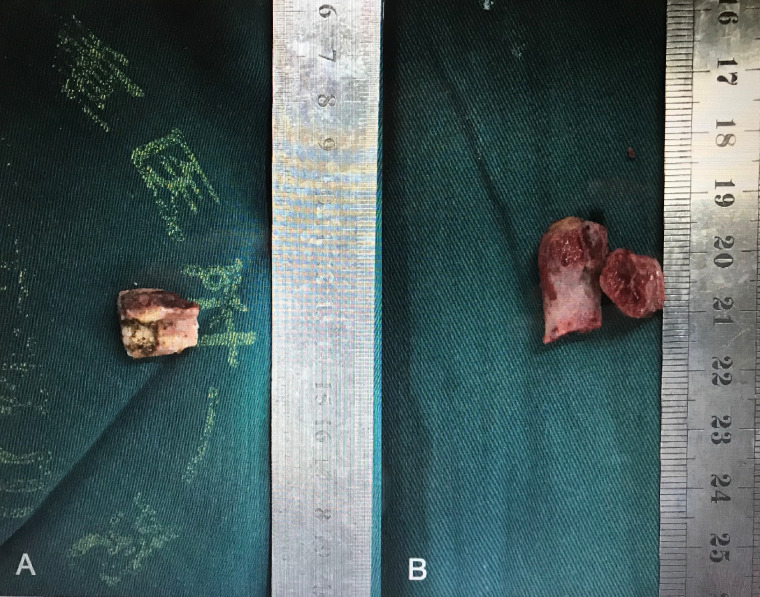
Photographs of trimmed LSP (A) and TPS (B).

**Table 1:** Information of patients

	Group A	Group B	Group C	P_AB_	P_AC_	P_BC_
No. of patients (n)	35	33	31			
Male/female (n)	19/16	21/12	15/16			
Mean age (years)	33.65±11.06	47.13±18.10	41.48±13.99	<0.0001	<0.0001	0.7619
Hospital stay (days)	14.05±3.58	17.61±5.03	17.88±10.06	<0.0001	<0.0001	0.9168
Surgery time (minutes)	182.40±23.82	205.10±25.60	231.40±80.90	0.6327	<0.0001	<0.0001
Blood loss (ml)	280.80±76.82	301.50±165.40	510.00±300.00	0.8305	<0.0001	<0.0001
Drainage (ml)	340.00±167.20	450.55±171.80	620.90±150.65	0.0176	0.0192	0.0444
Mean fusion time (months)	12.90±3.91	6.75±1.55	5.52±1.64	<0.0001	<0.0001	0.3465
Follow-up (months)	43.90 ± 10.39	45.30±6.20	44.32±7.17	0.1530	0.1760	0.9529

**Table 2:** Clinical and radiographic outcomes

Parameter	Group A	Group B	Group C	P_AB_	P_AC_	P_BC_
**ESR**						
before treatment	79.75±7.55	62.51±17.45	63.72±15.61	0.0126	0.0147	0.8867
Final FU	13.95±4.50	10.61±2.96	13.36 ±3.13	0.3044	0.9896	0.4096
**VAS**						
before treatment	6.95±0.94	6.5±0.75	6.68±1.25	0.8702	0.7670	0.8023
Final FU	1.95±0.69	1.58±0.95	1.68±0.76	0.8296	0.8888	0.9607
**ODI**						
before treatment	39.95±4.84	40.25±4.12	40.76±4.74	0.7247	0.7019	0.9400
Final FU	4.50±1.54	4.56±1.33	4.88±1.11	0.9417	0.9296	0.7670
**Segmental angle (^°^)**						
before treatment	17.97±2.80	18.77±2.49	24.61±14.96	0.6452	0.1638	0.5004
Final FU	16.25±3.64	15.01±3.55	17.62±5.38	0.7643	0.8090	0.7289
**Intervertebral height (cm)**						
before treatment	11.30±1.80	11.70±1.90	12.19±1.92	0.1377	0.1377	0.1962
Final FU	10.30±1.25	10.60±1.50	11.99±1.17	0.3329	0.1639	0.9010

ODI: Oswestry Disability Index; VAS: visual analog scale; ESR: erythrocyte sedimentation rate; CRP: C-reactive protein; FU: follow-up.

## Abbreviations

TB: tuberculosis
LSP: lamina with spinous process
TPS: transverse process strut
IG: iliac graft
ODI: Oswestry Disability Index
VAS: visual analog scale
ESR: erythrocyte sedimentation rate
CRP: C-reactive protein
FU: follow-up
ASIA: American Spinal Injury Association
HIV: human immunodeficiency virus
WHO: World Health Organization
